# Supplementary material for: Hope4Genes: a Hopfield-like class prediction algorithm for transcriptomic data
Source: Sci Rep. 2019 Jan 23;9:337. doi: 10.1038/s41598-018-36744-y (PMC6344502; doi:10.1038/s41598-018-36744-y)
Supplement: Supplementary file 1 — Supplementary Information [file 41598_2018_36744_MOESM1_ESM.pdf]

Hope4Genes: a Hopfield-like class prediction algorithm for transcriptomic data

Laura Cantini[1,2] Michele Caselle[3]

[1] PhD in Complex Systems for Life Sciences,University of Torino, Torino, Italy
[2] Institut Curie, PSL Research University, INSERM U900, Mines ParisTech, Paris, France
[3] Università degli Studi di Torino, Department of Physics and INFN, via P.Giuria 1, I-10125 Turin,Italy

Supplementary Table 1. The size of the gene signatures used for the five exmaples is here reported.

Example number	number of classes	size signature class 1	size signature class 2	size signature class 3	size signature class 4	size signature class 5
1	2 classes	19	16			
2	2 classes	202	552			
3	2 classes	801	445			
4	4 classes	388	667	174	374	
5.1	5 classes	956	125	387	291	134
5.2	4 classes	956	125	387	291	
5.3	3 classes	956	387	291		
5.4	4 classes	956	125	387	291	

Supplementary Table 2. Percentage of correctly classified samples of Hope4Genes, NTP, CART, WV, SVM and k-NN across the different examples.

Example number	number of classes	Hope4Genes	Hope4Genes FDR 5%	NTP	NTP FDR 5%	CART	WV	SVM	k-NN
1	2 classes	94%	100%	89%	100%	91%	97%	91%	94%
2	2 classes	86%	94%	88%	94%	92%	86%	80%	88%
3	2 classes	100%	-	100%	-	100%	83%	100%	83%
4	4 classes	100%	-	100%	-	69%	-	98%	94%
5.1	5 classes	56%	69%	58%	60%	53%	-	70%	53%
5.2	4 classes	70%	75%	69%	70%	63%	-	73%	56%
5.3	3 classes	70%	98%	68%	91%	60%	-	74%	77%
5.4	4 classes	65%	65%	62%	62%	63%	-	62%	62%
